# FindFoci: A Focus Detection Algorithm with Automated Parameter Training That Closely Matches Human Assignments, Reduces Human Inconsistencies and Increases Speed of Analysis

**DOI:** 10.1371/journal.pone.0114749

**Published:** 2014-12-05

**Authors:** Alex D. Herbert, Antony M. Carr, Eva Hoffmann

**Affiliations:** MRC Genome Damage and Stability Centre, School of Life Sciences, University of Sussex, Brighton, BN1 9RQ, United Kingdom; National Cancer Institute, United States of America

## Abstract

Accurate and reproducible quantification of the accumulation of proteins into foci in cells is essential for data interpretation and for biological inferences. To improve reproducibility, much emphasis has been placed on the preparation of samples, but less attention has been given to reporting and standardizing the quantification of foci. The current standard to quantitate foci in open-source software is to manually determine a range of parameters based on the outcome of one or a few representative images and then apply the parameter combination to the analysis of a larger dataset. Here, we demonstrate the power and utility of using machine learning to train a new algorithm (FindFoci) to determine optimal parameters. FindFoci closely matches human assignments and allows rapid automated exploration of parameter space. Thus, individuals can train the algorithm to mirror their own assignments and then automate focus counting using the same parameters across a large number of images. Using the training algorithm to match human assignments of foci, we demonstrate that applying an optimal parameter combination from a single image is not broadly applicable to analysis of other images scored by the same experimenter or by other experimenters. Our analysis thus reveals wide variation in human assignment of foci and their quantification. To overcome this, we developed training on multiple images, which reduces the inconsistency of using a single or a few images to set parameters for focus detection. FindFoci is provided as an open-source plugin for ImageJ.

## Introduction

The accumulation of proteins into cytologically-detectable foci is used as a phenotypic measurement in a wide range of biological applications. For example, the accumulation of the phosphorylated form of γH2AX is widely used as a biomarker for genotoxic insult, since it accumulates into distinct foci in the nucleus in response to DNA damage [1], [2], [3], [4], [5], [6]. The quantification of foci is often done manually, leaving the method open to inconsistencies and human error (*e.g.* [7]). Quantification and detection of foci may lead to different biological interpretations as well as a lack of reproducibility of biological results that is not due to true biological differences, but rather attributable to human error in focus detection. One particular concern with manual detection of foci is high variability between experimenters [7]. Often, the first image published and the quantification of foci of a particular protein becomes the ‘ground truth’ to which all subsequent studies are expected to adhere.

Improvement of consistency in focus detection can, in theory, be achieved by automated computational tools with parameterized algorithms. As a starting point, each pixel with higher values than all the surrounding pixels are candidate foci. This is, however, non-selective leading to undesired false foci that are not large enough, not of the correct shape or are artefacts of noisy data. To reduce the selection of false foci, parameters can be introduced that select characteristics such as height, size, shape and distance from other foci [8], [9], [10]. Such optimization of parameter settings is usually carried out manually for individual proteins in a labour-intensive fashion. Standardization of parameter settings is therefore only commonly employed to set up analysis pipelines, when a large number of samples will be analysed [10], [11]. Reproducibility of such analyses are also limited by the availability of commercial software [7]. With each additional parameter the software can be more specific at the expense of being less intuitive for the user (*e.g.* [12]). Ideally the algorithm should: (1) allow extensive sampling of all possible parameter combinations in an intuitive manner; (2) be fast enough to provide real-time results so that changes to parameters can be visualised [9]; and (3) support automated pipelines for batch analysis [10].

One important missing component of open-source focus identification software in biology is the possibility for users to train the detection algorithm to match or predict their assignments (machine learning). This would likely improve consistency of analysis in different images. Machine learning is used in a range of biomedical applications and can be used as predictive or detective tools that greatly enhance accuracy and reproducibility in a time-efficient manner [13], [14]. In microscopy, machine learning has been used to analyse a range of biological processes ranging from the detection of subcellular protein localization to the prediction of mitochondrial fission/fusion events [15], [16], [17], [18], [19], [20], [21], [22], [23], [24], [25], [26], [27], [28], [29], [30], [31], [32], [33], [34], [35]. Generic focus detection using machine learning has, however, not been developed yet. Being able to employ a training algorithm that experimenters can ‘train’ to match or predict their assignments without having to manually select or measure a vast list of poorly-understood parameters would allow users an intuitive approach to focus identification. Employing a training algorithm would also have the advantage of fast, repeatable focus detection.

Here, we identify four factors that influence consistency in focus selection between experimenters and provide open-source, freely available software that can be trained to closely match experimenters' patterns of focus detection. FindFoci allows individuals to train the algorithm to closely match their focus assignment using a small number of images and then apply the parameters across a large number of images. FindFoci facilitates transparency in the parameters used by different experimenters to detect foci and provides visual tools that can be used to compare experimenters' detection of foci. Parameters can be stored with images for future use, thereby facilitating best-practise for subsequent re-analysis by the same or different experimenters. The pipeline is fast, which allows a comprehensive parameter space to be searched and makes it time efficient compared to manual detection. Training on single images very closely matches manual assignment for that image, but application of the parameters across the dataset shows a large variation in the success rate of focus detection. We show that training the algorithm on multiple images significantly improves concordance between experimenters and is therefore preferable to manual focus detection or training on selected single images. Subsequent batch-analysis using the parameters derived from multiple images significantly improves consistency in image analysis compared to software where a range of parameters are selected manually by users without training the detection algorithm (*e.g.* CellProfiler [10]).

## Results

### Experimental set-up

Consistency in the identification of homogenous foci with high intensity and low background is straightforward. One way of achieving this is during the front-end processing of the biological samples, for example by treating with detergents. This, however, causes data loss of potentially significant biological importance. In this programme of work, we wanted to (1) explore the consistency of focus detection when foci are of heterogenous intensities, shapes, and sizes and when background noise is significant; (2) determine whether current standards of manually determining a range of parameters gives consistent focus quantification; and (3) assess whether machine learning can be used to mimic human assignments, thereby making focus quantification transparent, fast, and consistent.

**Figure 1:**
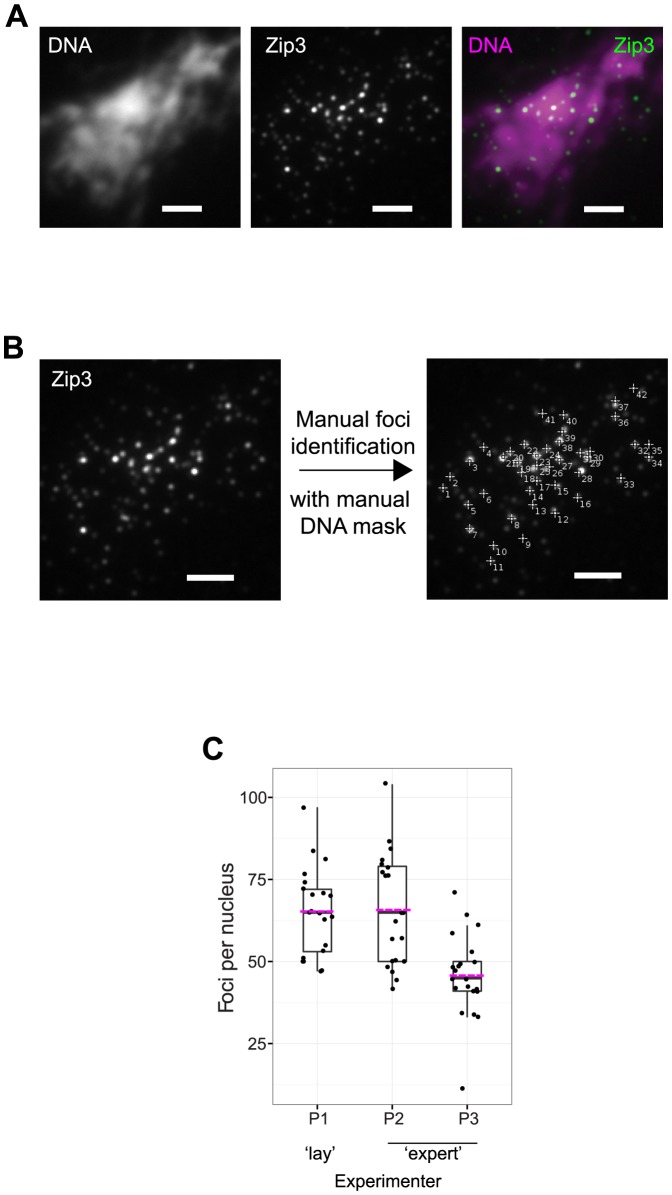
Example image and manual focus identification. (A) Spread nuclear DNA from budding yeast meiosis stained with DAPI (DNA, left) and antibodies against Zip3-GFP (middle). The merged image is shown in false colour on the right. Scale bar  =  2 µm. (B) Experimenter-labelled maxima (foci) of Zip3-GFP within the DNA region (determined by the experimenter). (C) Quantification of foci from the same 21 images by three different experimenters (P1 to P3). ‘Expert’ refers to scientists with previous knowledge of Zip3 or Msh4 quantification. Magenta bars represent the arithmetic mean, the black bar in the box-and-whisker plot shows the median value and all individual data points are shown as dots. Whiskers extend 1.5× of the interquartile range or to the minimum/maximum value, when these fall within 1.5× of the interquartile range.

To explore human variation in focus assignment and the sources thereof we used spread, meiotic nuclei from budding yeast stained with antibodies against two different DNA repair proteins, Zip3-GFP and Msh4-GFP. We chose Zip3 and Msh4 because both proteins have been quantified manually in several publications and varied numbers of foci have been reported [36], [37], [38]. 21 images of spread meiotic nuclei from budding yeast were imaged using fluorescently labelled antibodies staining for Zip3-GFP (images 1–14) or Msh4-GFP (15–21; all 21 images are available in Dataset S1). A representative example image is shown in Figure 1A, where the foci vary in intensity and size and the DNA region does not have a visually distinct boundary. Each image was analysed manually by three independent experimenters (P1–P3) using ImageJ to identify foci within the DNA region. Experimenters were asked to manually count the foci on the DNA, without being provided with prior knowledge of a DNA mask. No restrictions on how to determine background, brightness/contrast or other image parameters were set and experimenters were free to use any image visualization tools such as heatmaps and 3D relief maps to detect foci. Two experimenters had substantial knowledge of previously published data on the quantification of Zip3 and Msh4 foci (‘experts’). The third experimenter was a person without prior knowledge of Zip3 and Msh4 focus counts (‘lay’ person). The two experts were given the brief to count foci as part of their research. This allowed us to compare how researchers within the field count foci. The ‘lay’ person, without pre-conceived knowledge of Zip3 and Msh4 foci, was briefed to identify foci with a ‘significant’ (self-selected) intensity above background. All three participants were informed that two other experimenters were analysing the same image. This ensured that all participants counted as carefully as possible. An example of manual assignment of foci is shown in Figure 1B. In this example, there are several foci that were picked that may appear to be outside of the region of the DNA (Figure 1A, DNA). This is due to the fact that experimenters were free to assign where they considered the DNA to stop. In this case, all foci were indeed within the DNA mask, as determined using an Otsu thresholding algorithm (data not shown). This emphasizes the subjectiveness of the manual assignment task.

### Human variation in spot assignment

The manual assignments of foci in 21 images by the three experimenters resulted in an average number of spots per cell of 65, 66 and 46 (Figure 1C). Despite two experimenters being ‘experts’ in the field their quantification were significantly different from each other (p <0.001, Kruskall-Wallace). The ‘lay’ person and one of the experts appeared to detect similar numbers of foci (P1 versus P2, Figure 1C). These observations show that despite previous knowledge of the field, experts differ in their judgement of which foci to include in their quantification. Similar findings were reported for γH2AX [7].

To understand the nature of the different quantification, we compared the focus assignments between the experimenters by iteratively assigning the closest pairs of foci, up to a radius of 8 pixels. We chose 8 pixels because the average focus width was approximately 5 pixels, thus allowing experimenters to click on either side of the focus's maximum. Matched pairs and the remaining unmatched assignments were counted and match statistics between experimenters were computed as follows:
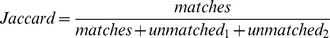





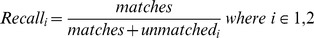


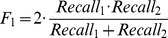



The Jaccard score is a measure of the overlap between the two sets, where a score of 0 indicates no overlap between the two datasets and a score of 1 indicates complete overlap or concordance. In contrast, Recall is a measure of how much one set overlaps with another. Recall_1_ measures the fraction of foci selected by one experimenter that matched those of a different experimenter. For example, experimenter P1 versus experimenter P2. Conversely, Recall_2_ measures what fraction of foci selected by the second experimenter matched those of first experimenter. In this case experimenter P2 versus experimenter P1. The two Recall scores can be combined using an equal weighting to yield the F1 score. Both the Jaccard score and the F1-score range from 0 to 1 and evaluate the agreement between the two experimenters. A score of 0 indicates no similarity and a score of 1 demonstrates 100% similarity. The average Jaccard scores between pairwise experimenters were 0.725 (P1 versus P2), 0.679 (P1 versus P3) and 0.680 (P2 versus P3). The average F1-scores were 0.834, 0.790 and 0.798, respectively (Table 1).

**Table 1:** Comparison of manual focus selection by two different experimenters across 21 images^a^.

Comparison		Jaccard			F1-score		
Experimenter 1	Experimenter 2	Raw	Aligned	Change (%)	Raw	Aligned	Change (%)
P1	P2	0.725	0.734	1.27	0.834	0.839	0.54
P1	P3	0.679	0.679	0.03	0.790	0.789	−0.13
P2	P3	0.680	0.687	0.98	0.798	0.804	0.68

a Matches were iteratively assigned using the closest pairs within 8 pixels. The table shows the comparison of the raw spot assignments and the aligned spot assignments where points are moved to their closest local maximum (see methods for details). Scores are averaged over 21 images.

**Figure 2:**
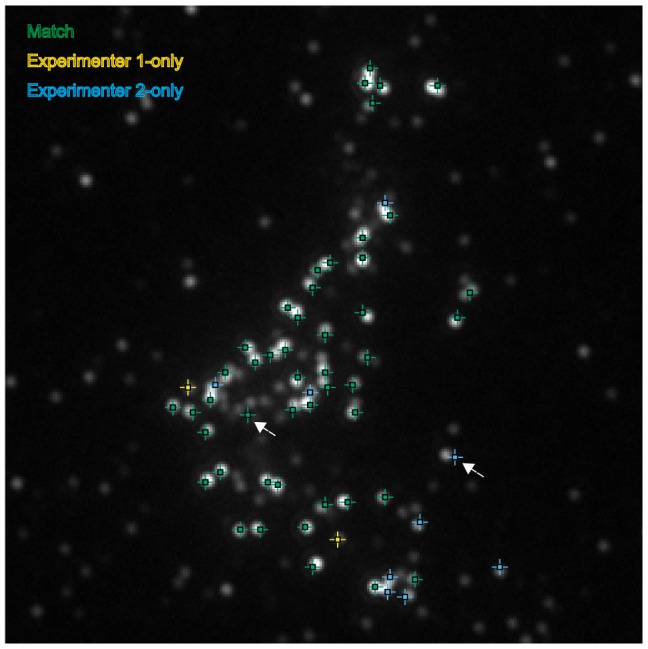
Comparison of focus assignments between experimenters. An example image shows matches (within 8 pixels) of two experimenters in green using the position of experimenter P1. Unmatched foci that were selected only by experimenter P1 are shown in yellow and unmatched foci selected only by experimenter P2 are indicated in blue. The Jaccard score for this comparison was 0.81. Only spots within the DAPI stained region were extracted for analysis. Arrows indicate examples where experimenters have clicked a short distance from a maximum.

Using an algorithm that aligns the selected position to the closest local maximum (focus) only marginally improved the Jaccard and F1-scores (see below). This suggests that setting the 8 pixel comparison distance between any two experimenters adequately allows for variability in the position within a specific focus that the experimenters click to select it (*e.g.* Figure 2, arrows). Overall, the Jaccard values and F1-scores suggest that 20–30% of focus assignments are unmatched between any given two experimenters.

### Four identified sources due to human error and personal interpretation cause variation in focus assignment

**Figure 3:**
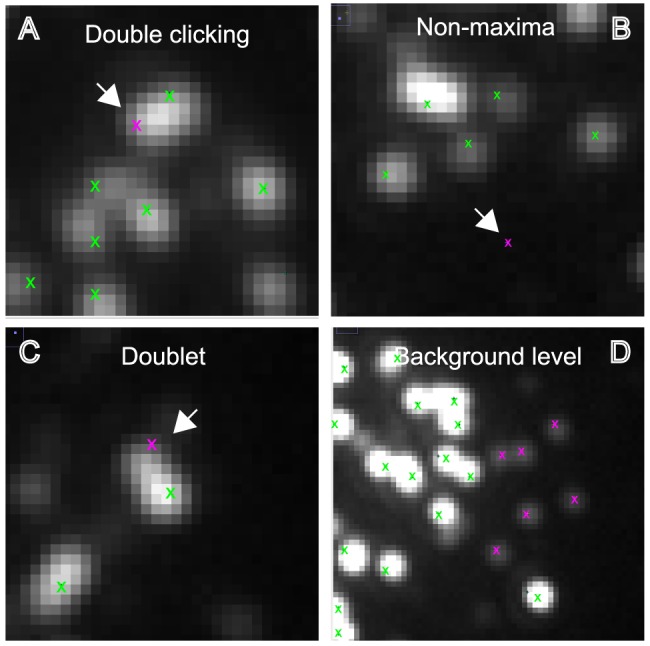
Sources of inconsistency in focus selection between experimenters. Matches are shown in green and unmatched points in magenta. (A) Dual assignment of a single maximum by experimenter 2 (‘double clicking’); (B) mislabelling of non-true maxima; (C) interpretation of diffraction-limited foci as a single focus or dual foci (‘doublet’); and (D) arbitrary selection of different background levels to determine inclusion of foci in the analysis. Arrows indicate the discordant foci in magenta.

To understand the differences between experimenters in focus assignment, the matched and unmatched foci were visualised by superimposing the assignments from each experimenter on the same image (Figure 2). Matches were labelled in green and unmatched points from each set in either blue or yellow. Visual inspection of the images revealed at least four sources of mismatches. First, we observed a few cases of dual assignment (clicking the same spot twice; Figure 3A, matches in green, assignment by a single experimenter in magenta); or experimenters selecting a region without any local maxima (Figure 3B). The two main factors influencing the focus quantification, however, were interpretation of diffraction-limited foci as doublets (Figure 3C) and setting different background levels (Figure 3D). Both of these were based on experimenters' personal interpretation. For example, experimenter P2 selected many incidences of ‘doublets’ compared to P1 and P3. In contrast, P1 set a low background level compared to P2. Thus, although P1 and P2 had similar overall focus counts (Figure 1C), they arose from very different methods of selecting foci for inclusion in the quantification. P3 set the highest arbitrary threshold for ‘background’ noise, removing a large number of ‘faint’ foci (*e.g.* Figure 3D, magenta). This resulted in a the lowest mean count of foci (Figure 1C).

### Low intensity foci cause discordance in quantification between experimenters

**Figure 4:**
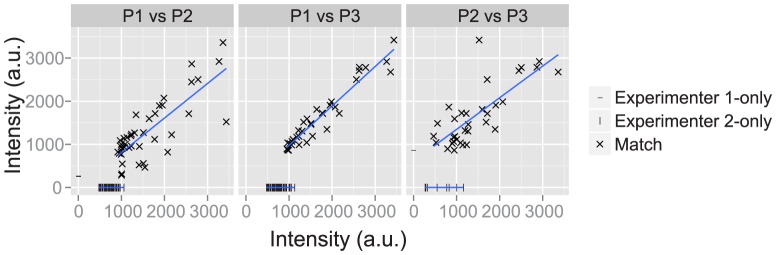
Interpretation of low intensity foci causes variation in focus quantification between experimenters. Plotted are the pixel intensity of the foci selected by experimenter P1 and P2 (left panel); P1 and P3 (middle panel); and P2 and P3 (right panel) for an example image from the dataset. Foci that were selected by both experimenters (within 8 pixels of each other) are shown as crosses (‘Match’); foci selected by experimenter P1 only are shown as a dash on the X-axis; and foci selected by experimenter P2 are shown as a dash on the Y-axis. A best fit line for the matched pairs is shown in blue.

If experimenters differ in their assessment of background noise, then one would expect faint foci to cause discordance between experimenters, whereas high-intensity foci should be picked by both experimenters in the three pairwise comparisons. To quantify whether this was the case, we plotted the intensity of the marked pixels from each experimenter in a scatter plot (a plot for a typical image is shown in Figure 4). Matches are marked with a cross; unmatched points are marked using a single intensity value and are placed on the X or Y axes for each experimenter, respectively. The majority of unmatched points on the X and Y axes are in the lower range of the pixel values (Figure 4). This shows that unmatched foci selected by only one of the experimenters tend to be the less intense maxima. A best fit line is shown for the intensity of matched pairs between experimenters (Figure 4, blue lines). Deviation from the line indicates variation in the marked centre of the focus, which should be the identical maximal value for the spot. This highlights inaccuracy in one or both experimenters in selecting the pixel with the maximal value in the focus.

**Figure 5:**
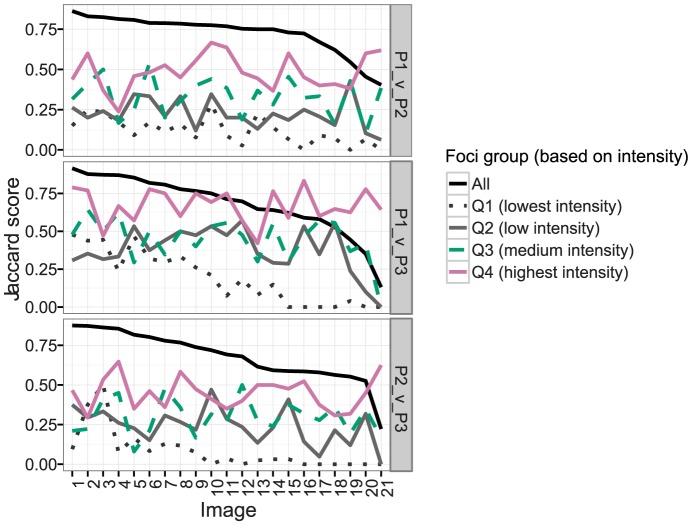
Agreement of focus selection between any two experimenters decreases with reduced focus intensity. Foci were arranged by pixel intensity and divided into quartiles. The match statistics (Jaccard scores) were computed for each quartile (Q1–Q4) and the entire set (‘All’). Images are arranged in descending order of overall Jaccard score. Q1 consisted of the 25% of foci with the lowest intensity, Q2 the 26^th^–50^th^ percentile (‘low intensity’); Q3 the 51^st^–75^th^ percentile (‘medium intensity’), and Q4 the 25% of foci with the highest intensity (‘highest intensity’).

To investigate the effect of pixel intensity on matched foci, the union of the two sets of foci was ranked by intensity values and divided into quartiles (Figure 5; images arranged by overall Jaccard score). The match statistics for each quartile were computed individually. Figure 5 shows the Jaccard score for each quartile over all of the images in the dataset for each pair of experimenters. The trend was for the lower quartiles (Q1 and Q2), which have lower intensity foci, to have a lower score than the upper quartiles (Q3 and Q4). Thus, the less intense foci are less likely to be selected by both experimenters. Consistent with this, the average Jaccard score of each successive quartile increases (data not shown) indicating that the less intense spots are where the differences between experimenters are more pronounced.

**Figure 6:**
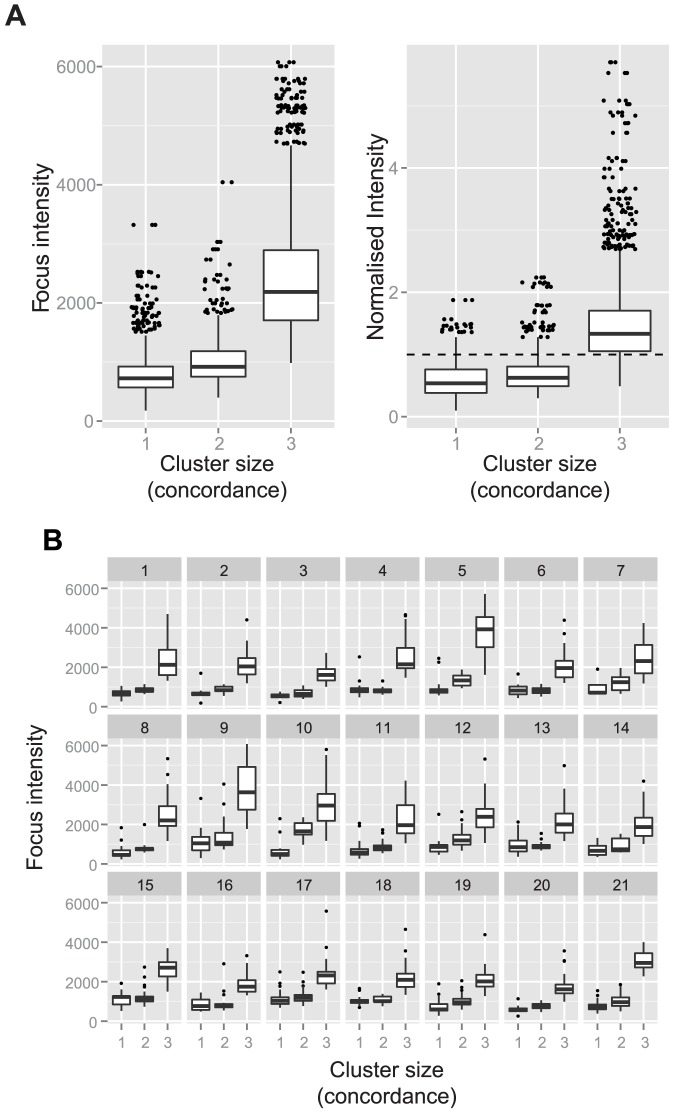
The average focus intensity is higher with increasing agreement between experimenters (cluster size or concordance). Greedy clustering was performed by first comparing foci selected by two experimenters using an 8 pixel radius. When the focus was selected by both experimenters, a cluster of size 2 was generated. Foci that were only picked by a single experimenter were used to seed a cluster of size 1. The clusters were compared to the third experimenter and any matches used to increase the cluster sizes such that matches between all three experimenters gave rise to cluster size 3. Clustering was performed using experimenter order 123, 231 and 312 and all results combined for plotting (A). Because experimenters clicked on slightly different parts of the focus, the focus intensity was calculated as the average pixel value selected by the experimenters. The normalized values for the cluster were calculated by dividing the average pixel value by the median value of all of the selected foci from the same image. The horizontal, dashed line indicates a normalized value of 1. The analysis of individual images are shown in (B).

Pairwise analysis between experimenters was complemented with a clustering analysis of the assignments between all three experimenters. We used a centroid linkage clustering algorithm to count foci that were selected by all three experimenters (Figure 6). Briefly, the matches between two experimenters were used to create clusters (size 2) using the average coordinates. The unmatched foci were used to create clusters of size 1. The clusters were compared to the third experimenter resulting in clusters of size 3 (all three experimenters picked the focus), size 2 (two experimenters picked the focus), or size 1 (a uniquely identified focus by a single experimenter). To avoid bias, we repeated this process three times, using all possible initial combinations of experimenters. Figure 6A shows a box plot of the raw and normalized intensity values of the foci for each cluster size (‘concordance’) for all 21 images combined. From this, it is clear that the intensity of foci selected by all three experimenters is higher than those selected by two or one alone. Furthermore, analysis of individual images shows that this conclusion applies consistently across all of the 21 images (Figure 6B). We conclude that the intensities of foci are higher for larger clusters indicating there is more agreement between the experimenters on the high intensity foci.

### An automated focus identification algorithm can be trained to match human assignments

Our analysis of the variation in experimenters' manual assignment of foci led us to develop an automated training algorithm with three defined goals. First, we sought to understand how different people are assigning foci, thus the algorithm should contain descriptive parameters that can be easily understood. Second, the algorithm should allow improved consistency of focus assignment across different experimenters, in essence eliminating human errors. Third, the algorithm should be fast and easily applied by biological users to produce transparent outputs.

To this end, we created FindFoci, an open access, freely accessible and intuitive plugin for ImageJ/ImageJ2. In essence, it allows users to select a representative (or multiple representative) image, mark the foci, and use this to train the algorithm to find the optimum parameters for focus selection. Parameter combinations are assessed using the Jaccard or F1-score against the manually-selected foci. The parameter combination generating the best score can then be applied to many different images (batch mode). The outputs include marked-up (or annotated) images of selected foci, masks of foci with different rendering, as well as an extensive table of results.

The focus-finding algorithm identifies and expands local maxima using a downhill gradient to mark all pixels assigned to each focus. The algorithm allows foci to be filtered using parameters such as area, intensity, and height above the saddle points (with neighbouring maxima) or the height above background. To train the algorithm, the FindFoci Optimiser enumerates a range for each parameter and determines the best combination of values of the included parameters given a representative image. In processing each image, rather than analysing each combination of parameters individually, the optimiser caches staging points (*i.e.* stores intermediate results) to avoid repeated processing of duplicate parameters. For example, the algorithm identifies all foci, saves the intermediate results, then processes those results using filters for different focus size, height, etc. This staging avoids having to recalculate the results that would be unchanged for each new combination of parameters, thus increasing speed of analysis. In this study, we used 19,800 combinations of parameters, with an average run time of 49.5 ± 8.6 (SD) seconds *per* image (or 2.5 milliseconds *per* parameter combination). If any of the parameters from the highest scoring combination are at the boundary of the input range, then the optimiser will recommend expanding the range. This ensures the search space for the parameters is adequately sampled during the training of the algorithm.

In order to assess the performance of the algorithm in predicting human assignment of foci, we trained the algorithm on all of the manually-labelled images from each experimenter (3 experimenters, 21 images per experimenter, 63 in total). The training of the algorithm produces a ranked list of parameter combinations for each image. The ranking is determined by the Jaccard or F1 score for the foci identified by the algorithm compared to the manually-selected foci from the image on which the algorithm was trained. The top score for each image was averaged across the 21 images from each experimenter (Table 2, ‘Original’). Note that for each image the optimal parameter combination may differ. The average F1-scores per experimenter were 0.967 (P1), 0.933 (P2), and 0.958 (P3). This shows that the algorithm is able to closely match the human assignments on a *per* image basis.

**Table 2:** The FindFoci algorithm closely matches human assignments^a^.

Experimenter	Original	Aligned	Change (%)
P1	0.967	0.972	0.483
P2	0.933	0.944	1.175
P3	0.958	0.963	0.608

a The FindFoci algorithm was trained on individual images and the highest F1-score reported and averaged over the 21 images per experimenter. The analysis was performed using the original selected pixel coordinates (‘Original’) or the coordinates after alignment to their appropriate local maximum (‘Aligned’).

### Parameterized focus detection reveals human inconsistency in focus assignment across images

Next, we sought to assess whether the optimal parameter combination obtained from a single image is able to detect foci accurately from other images, either from the same experimenter or from images marked by different experimenters. To this end, we trained the algorithm on a single image to match the foci identified by one experimenter and then used the parameter combination to predict foci for all 21 images. These predicted foci were then compared to the manually-detected foci from each experimenter (*i.e.* 3 × 21) using the F1 score. This process was repeated for each of the manually-labelled images, resulting in 63 × 63 (3969) pairwise comparisons.

**Figure 7:**
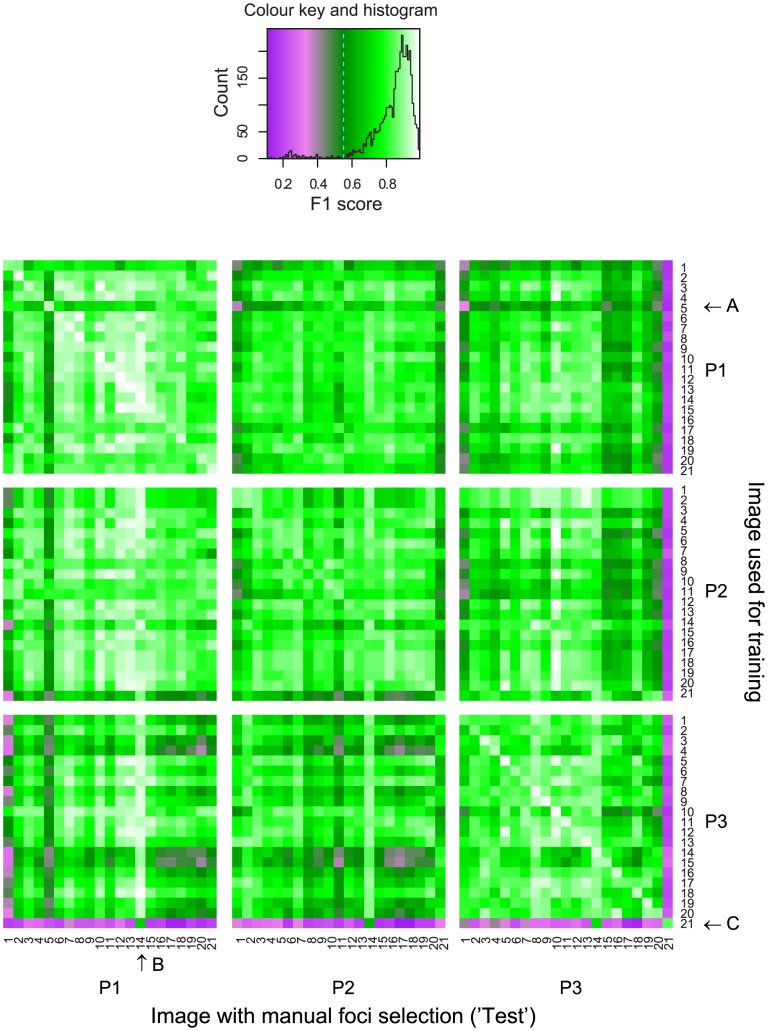
Parameters obtained from a single image show wide variation in performance when applied across the entire dataset. The heatmap shows the F1 scores for the optimal parameters from the training image (‘Image used for training’) applied to all 21 assigned images from each experimenter (‘Image with manual focus selection’ (‘Test’)). P1, P2, and P3 refer to the experimenter ID. Arrows labelled A to C are discussed in the main text. The colour key and histogram of all F1 scores are shown above the heatmap.

Figure 7 shows a heatmap of the F1 scores when comparing the foci obtained using each of the 63 optimised parameter sets (‘Image used for training’) to every other manually-labelled image (‘Image with manual focus selection’ (‘Test’)). F1 scores close to 1 are show in light green and white, whereas scores below 0.4 are shown in magenta. Horizontal lines show how well a single set of parameters that have been derived from one image apply across all of the manually-scored images. For example, when image 5 from experimenter P1 was used to train the algorithm, the parameters obtained predicted the manual selection on the other 62 images very poorly (arrow labelled ‘A’). This may indicate that the experimenter has changed her/his assignment method for a certain image or the image may be non-representative of the dataset. For example, the background surrounding this nucleus may be higher.

Vertical lines show how well foci selected from a single manually-scored image is predicted by every set of optimal parameter combinations from the 63 training images. For example, there were several examples of high scoring as well as low scoring vertical lines. The high scoring vertical lines indicate that parameters obtained from other images were equally applicable to the image (*e.g.* image 14 from experimenter P1, arrow labelled ‘B’). These high scoring images suggest that the foci may be very strong and easy to recognize. The low scoring vertical lines show examples where the experimenter has used a very different style of focus selection compared to the other experimenters. For example, images 15 to 21 from experimenter P3 showed low F1 values when compared to training from other images. When used for training, image 21 from experimenter P3 also poorly predicted foci in all other images (arrow labelled ‘C’). Visual inspection of these foci selected by experimenter P3 suggested that the image may have been partially or incompletely scored. Thus, although the algorithm matches P3's assignments for images 15-21 well, P3's selection of foci in these images is clearly different from those of experimenters P1 and P2.

The central diagonal running from the upper left to the lower right corner indicates the optimal score for each image (Table 2, ‘Original’). These occur when the same image is used for both training and testing, *i.e.* the maximum score when training the algorithm on that image. The central diagonal was consistently high for experimenters P1 and P3 but less pronounced for experimenter P2, indicating that the algorithm is less able to match P2's method of assignment. This is likely due to the selection of doublets by P2 (Fig 3C), which are not chosen by the algorithm.

The majority of the points, *i.e.* those that are not on the central diagonal (also referred to as ‘off-diagonal’), indicate how successfully the parameters from one image can be applied to another. These off-diagonal points had lower F1 scores compared to the central diagonals (especially for P3), suggesting that the experimenters were picking foci with different parameters in the 21 images. Although P2's central diagonal scores were lower than P1's and P3's, training on P2's images gave good concordance across other images from the same experimenter as well as P1. Indeed, some of the training on P2's images gave higher concordance with P1's images compared to other P2's images within the same set. Thus, although the algorithm is less capable of matching P2's focus assignments, the parameters provided by training are applicable to other manually-scored images. Finally, P3's parameters were poorly applicable to P1 and P2 suggesting a very different style of scoring foci. In particular images 3, 4, 14, 15, and 21 trained by P3 gave poor F1 scores on the images from P1 and P2 (dark-green/purple horizontal lines; arrow labelled ‘C’, Figure 7).

If experimenters scored the same foci in the same image, then one would expect a central diagonal in each of the nine squares. Although central diagonals were observed within experimenters, they were not present between experimenters. This indicates that the parameters for one image are no more applicable to the same image or to a different image, when two different experimenters are compared. This supports the notion of highly variable, inconsistent, subconscious ‘parameter’ usage by experimenters when manually analysing images. This concept is further supported by the absence of high scoring (white) horizontal lines, *i.e.* no image on which the algorithm was trained gave particularly high performance across all other images.

The dataset in which Msh4-GFP foci were picked (images 15–21) yielded particularly low F1 scores, both when trained on P3's focus selection (horizontal lines) or when trained on P1's or P2's images and subsequently tested against P3's manual assignments (vertical lines). A non-parametric ANOVA analysis revealed that this dataset (Msh4-GFP foci) contributed substantially to the variation in the F1 scores (13%; p <0.001) suggesting that images from this dataset were particularly variable. Further analysis revealed that image 21 analysed by experimenter P3 caused the majority of variation in focus assignment (10%). This is the image that was incompletely annotated (see above). When image 21 was removed from the analysis, the contribution of the Msh4-GFP dataset was no longer significant. This shows that using parameters optimised on a single image may cause severe discordance in identified foci when applied to other images. In summary, the algorithm can be trained to replicate focus identification on every image. However, variation within and between the F1 scores when the parameters from a single image are applied to other images reveals substantial variation on an image-by-image basis.

### Automated training across multiple images from one experimenter increases consistency when compared to other experimenters

**Figure 8:**
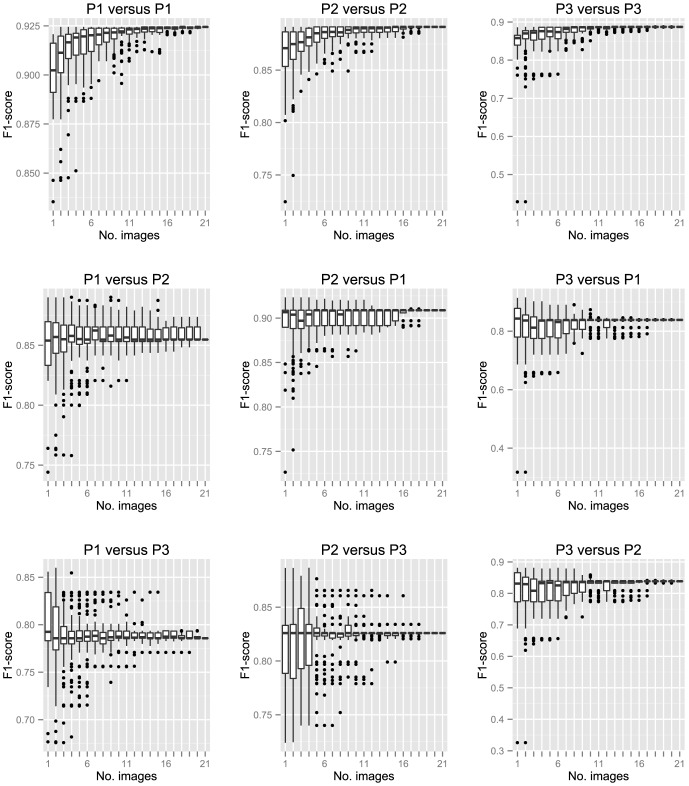
Training on multiple images improves consistency in focus detection. The FindFoci Optimiser was trained using 1 to 21 images. When the number of combinations of images used for training was small (n = 1 or n = 20 has 21 possible combinations; n = 2 or n = 19 has 210 possible combination) all possible combinations were used. For larger numbers of possible combinations, a random subset of 100 combinations were used to select images for training of the algorithm. The parameters obtained from training were tested against the manually marked images from each experimenter and the F1-score calculated. P1 to P3 refers to experimenter ID, with the first mention indicating the images used for training and the second referring to the manually-assigned images against which the parameter combination was assessed. *I.e.* ‘P1 versus P2' means that P1's marked images were used for training and the parameters were used to predict focus selection by P2.

Clearly, using parameter settings from a single image to analyse other images as part of a larger dataset may lead to significant variation (Figure 7). We next sought to determine whether the inconsistency of using parameter combinations derived from training on a single image could be reduced by training on multiple images. To do this, we created a plugin that allows training on multiple images. We trained the algorithm on possible combinations of 1 to 21 images from each experimenter and tested the derived parameter combinations on each of the manually-derived images. When the algorithm was trained on a single image (21 possible combinations) or two images (210 possible combinations) from one experimenter, we used all possible combinations of images to train the algorithm. When the number of combinations of images used for training was large (*e.g.* for n = 11 there are 352,716 possible combinations), we randomly sampled 100 unique combinations to be used for training. In Figure 8, we have plotted the average F1 score of parameters obtained from each combination of images when tested against 21 manually-marked images from the same experimenter (*e.g.* P1 versus P1; Figure 8 upper row) or against images from a different experimenter (*e.g.* P1 versus P2; Figure 8- rows 2 and 3). *I.e.* when a single image or 20 images were used to train the algorithm, 21 F1-comparisons to manually-selected images were obtained. The average F1-score per parameter combination (in this case 21) was plotted. When two images or 19 were used to train the algorithm, 210 average F1-scores obtained from prediction across the 21 manually-labelled images were obtained; for n = 3 to n = 18, 100 average F1-scores were plotted; and for n = 21, one F1 score was obtained.

We observed three trends. Firstly, training on an increasing number of images improved the consistency in all nine comparisons, as shown by the reduced interquartile range and reduction in outliers above and below the median (Figure 8, 9 individual plots). Thus, training on multiple images avoids skewed or biased batch analysis from parameter combinations derived from a single image, as demonstrated in Figure 7. Secondly, when the algorithm was trained on a subset of the test images (P1 versus P1; P2 versus P2; and P3 versus P3; first row Figure 8), increasing the number of images used in training significantly improved the median F1-score and reduced the number of outliers. In contrast, the median remained fairly stable, but outliers were greatly reduced when experimenters were compared against each other as a function of the number of images used to train the algorithm (Figure 8, rows 2 and 3). Visual inspection of the data in Figure 8 suggests that the majority of variation (*i.e.* outliers) are removed when roughly half of our dataset is used to train the algorithm. In the majority of cases, even using only five images reduces the low-scoring outliers significantly. Only in the case of comparing P1 or P2 against P3 is a larger number of images required to reduce the extreme outliers. This is due to the different focus selection method used by P3 in images 15–21 (Figure 7). Finally, when trained on all 21 images, the average F1 value when comparing experimenters increased up to 9% (Table 3, % Change). We conclude that training on multiple images reduces the risk of bias from using a non-representative image (or images) for training. Reduction of bias by using an automated algorithm trained on multiple images can explain the increase in F1 scores between experimenters compared to their manual assignments.

**Figure 9:**
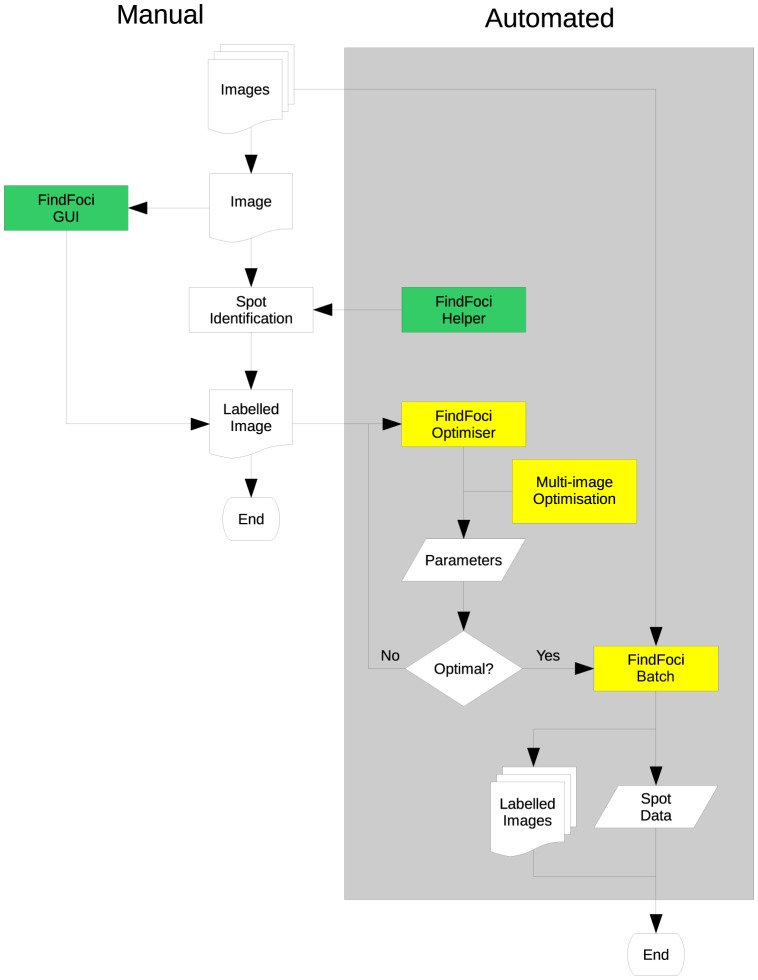
Flow chart of the automated workflow provided by the FindFoci software. Interactive tools are shown in green; automated scriptable tools are shown in yellow. After images have been collected, manual assignment of foci can be improved by the FindFoci Helper, which aligns clicked points to their true maximum. The FindFoci Optimiser can then be trained on the resulting labelled image to identify the best parameters for the algorithm. The FindFoci GUI provides a real-time update of the results while the user changes the parameters. Training on multiple images can be achieved using the Multi-Image Optimiser plugin in order to improve consistency. The number of initial images used for training is discussed in the main text. The parameters can be applied to a large set of images using the FindFoci Batch plugin.

**Table 3:** Comparison of F1-scores from human assignments or automated focus assignment to other experimenters^a^.

Training Set	‘Ground truth’	Original	Algorithm	Change (%)
P1	P2	0.834	0.855	2.45
P1	P3	0.790	0.786	−0.51
P2	P1	0.834	0.909	8.93
P2	P3	0.798	0.826	3.46
P3	P1	0.790	0.839	6.18
P3	P2	0.798	0.839	5.06

a The average F1 score between experimenters across 21 images using the original manually assigned foci from each experimenter (‘Original’) or the automated focus assignment trained across a dataset (‘Algorithm’) when compared against the ‘ground truth’, defined by another experimenter. Matches were iteratively assigned using the closest pairs within 8 pixels. Values are shown to three significant figures.

### Spot alignment before training improves the performance of the training algorithm

Human assignment of foci is biased towards picking bright spots in an image, which is subject to error due to the large dynamic range of the image that cannot be effectively viewed by the human eye. Visual tools such as heatmaps and 3D relief maps of intensities can aid the visualization of the dynamic range. However, ultimately, contrast and background subtraction is subjective and tends to be adjusted to favour either bright or low-intensity spots. Subjective adjustment of contrast invariably leads to pixel saturation in the displayed image (Figure 3D) and an inability to select the true maxima in a subset of foci. We therefore investigated whether the inconsistencies of assignments between experimenters could be reduced by semi-automated assignment, where foci selected by the experimenter are aligned to their nearest local maximum. For each image all of the potential maxima were identified and the surrounding pixels assigned to each maximum using an uphill gradient algorithm (see Materials and Methods). The manual assignments from each experimenter were processed in order of intensity, aligning each to their true maximum using the assigned maximum for the selected pixel. Only one point could be aligned to each maximum. In the event that the maximum had already been used the second point was aligned to the highest available maximum within a search radius. The radius was set as the distance to the unavailable true maximum thus searching for other candidates within an equivalent error margin. Foci without an available maximum were unmoved.

To prevent alignment to insignificant maxima (*i.e.* background noise), the alignment was not allowed if the new maximum was below a threshold height. This threshold was defined using all of the heights from the aligned maxima. Thresholding methods were: the bottom N^th^ percentile; 1.5× the interquartile range below the Q1–Q2 boundary; the mean of all of the heights for the aligned maxima minus a factor, which was multiplied by the standard deviation of the summed heights; and an adaptive threshold set by lowering the height from the highest point until the number of missed maxima above the threshold was a set fraction of the total maxima (missed and aligned). The last method relies on the human assignments including the most intense maximum in the image, *i.e.* 0% of maxima are missed when the threshold is the intensity of the highest maximum. This was the case for our dataset.

After thresholding, the alignment was assessed by comparing the aligned spots to all of the potential maxima above the height of the lowest aligned focus. F1 statistics were computed and used to assess the best method for aligning foci. The F1 scores were maximal when the adaptive thresholding method was used, with an allowed missed fraction less than 15% of the total maxima. *I.e.* clicked points are aligned to their local maximum and an intensity threshold for accepting the new maxima is set where at least 85% of true maxima would be detected. This prevents alignment of clicked points to insignificant, low intensity maxima. When using the optimal method, the average distances that foci moved were 0.767, 1.77 and 0.953 pixels for each experimenter, respectively, indicating that most foci chosen were close to their true local maximum.

The aligned assignments were then compared between experimenters by iteratively assigning the closest pairs up to a radius of 8 pixels. The average Jaccard score improved 1.27, 0.026 and 0.098 percent when compared to the unaligned assignments and the F1 score changed 0.54, −0.13 and 0.68 percent (Table 1). This indicates that the comparisons between experimenters' assignments were not greatly altered by the point alignment, likely due to the generous 8 pixel comparison radius applied.

We subsequently addressed whether prior alignment would improve the automated detection of foci on a *per* image basis. To assess this, the algorithm was retrained on each image using the aligned assignments for each experimenter. This improves the average F1 score 0.61, 0.48 and 1.2 percent when compared to the unaligned assignments (Table 2). Thus, although the F1 scores were already very high, indicating that the algorithm was able to closely match human assignments, alignment to local maxima prior to training nevertheless improved the ability of the algorithm to predict human focus selection.

To understand whether the prior alignment to local maxima would improve automated assignment of foci between experimenters, the algorithm was retrained across the dataset using the aligned assignments with the highest combined F1 score used to set the optimised parameters. As shown in Table 4, training on the aligned images results in higher scores compared to the unaligned images for each experimenter. This is not unexpected given that alignment of spots will ensure more spots are positioned on a maximum, increasing chances of matching the results obtained by the algorithm. More critically, when the optimal parameters from one experimenter are applied to another's assignments the scores are improved compared to training on unaligned images in all but one case. This demonstrates that alignment of foci before training improves the performance of the training algorithm.

**Table 4:** Focus alignment prior to training of the algorithm improves the F1-score^a^.

Training Set	Test Set	Raw	Aligned	Change (%)
P1	P1	0.925	0.930	0.581
P1	P2	0.855	0.863	0.963
P1	P3	0.786	0.787	0.151
P2	P1	0.909	0.915	0.654
P2	P2	0.891	0.893	0.138
P2	P3	0.826	0.820	−0.669
P3	P1	0.839	0.845	0.725
P3	P2	0.839	0.839	0.024
P3	P3	0.887	0.889	0.209

a Parameter combinations for the detection algorithm were optimised for all images in a given dataset (‘Training Set’). The F1-scores when the parameter combinations were applied to the same or other manually-labelled images (‘Test Set’) were averaged. Training was performed using the manually-detected foci (‘Raw’) or after the manually-selected foci were aligned to their appropriate local maximum (‘Aligned’). Values are shown to three significant figures.

### Automated training of FindFoci outperforms focus detection by an optimised batch-analysis (‘pipeline’) in CellProfiler

The current state-of-art to identify foci consistently is to set up a workflow of software algorithms to detect foci using the same parameter combination applied to all images. The parameters must be selected appropriately for the image analysis task. This is done by either estimating or directly measuring certain parameters (*e.g.* size of foci) manually. To evaluate the FindFoci algorithm against the current state-of-the-art, we compared the performance of the FindFoci algorithm to the object identification module of CellProfiler. CellProfiler is a configurable tool that allows image processing pipelines to be created from modules and then batch applied to images. Since CellProfiler cannot be trained automatically to optimize parameter settings, a pipeline for focus identification was constructed based on the pipeline developed by González *et al.* (2012) and manually optimized to achieve the best performance (see Materials and Methods). Our pipeline was trained manually for each experimenter using all 21 images. Parameter optimization was achieved by manually adjusting parameters until the highest average F1 score across images was obtained. Specifically, to ensure fair comparison between FindFoci and CellProfiler, we applied the same region of interest (DNA mask) to extract the foci for comparison. Performance was assessed by comparing the extracted foci to those identified manually by the experimenters. The pipeline using CellProfiler achieved an average F1 score across images of 0.655 to 0.792 (Table 5) showing reasonable agreement between the manually-selected foci and those detected by the optimized CellProfiler pipeline. The number of manually-selected foci that were identified by CellProfiler (Recall_1_) was high. However, CellProfiler also identified a large number of foci that were not selected manually by the experimenter (equivalent to Recall_2_, also known as ‘precision’). Thus, CellProfiler overpredicts foci, *i.e.* it identifies too many foci that are not real, based on human assignment.

**Table 5:** FindFoci outperforms CellProfiler^a^.

Experimenter	CellProfiler^a^	FindFoci	Change (%)
P1	0.792	0.925	16.73
P2	0.723	0.891	23.28
P3	0.655	0.887	35.48

a CellProfiler was manually trained by adjusting the parameters of the EnhanceOrSuppressFeatures and IdentifyPrimaryObjects modules until the best overall F1-score was achieved (see Methods for parameter ranges). FindFoci was optimised for all images in the dataset using the FindFoci Optimiser (Table 4, ‘Raw’). The average F1-score is shown for each dataset.

Comparison to the FindFoci algorithm revealed that the FindFoci method consistently outperforms the CellProfiler pipeline (Table 5). This is especially noticeable for Experimenter 3 with an increase of 35% in the F1 score. The performance improvement is attributed to two factors: (1) the automated training of the algorithm is able to process a far larger range of parameters than the manual training required for CellProfiler, thereby achieving a higher optimum; (2) the algorithm is more suited to describing the type of foci in this study. In addition it is noted that we are not experienced users of CellProfiler and a better pipeline may be possible. However this highlights the need for algorithms that can be trained on example datasets without expert knowledge of the software.

## Discussion

### Human variation in focus detection influences biological interpretations

Consistent and accurate focus detection is paramount to the study of biological systems. The preparation of samples as well as focus quantification are two critical factors to understanding the underlying biology. Quantification is particularly problematic because this is often carried out manually, sometimes without a record of images on which to compare different experimenters or without specification of parameter settings used to detect foci. The standard solution to this known variability is to manually select a range of parameters and analyse all images within a dataset with one specific parameter combination (*e.g.* FociCounter [12] and CellProfiler). In some cases, this improves consistency [7] as well as speed of analysis [11], but is ultimately highly dependent upon the sample preparation being consistent. For example, this could be achieved by reducing background noise levels from non-specific antibody staining within and between different slides.

In this work, we set out to determine what causes variation in focus detection between images and between experimenters. Moreover, we wanted to develop an automated training algorithm (FindFoci) that could predict experimenters assignment of foci and produce transparent outputs in real time. We reasoned this would allow experimenters to accurately quantify many different types of proteins without extensive investment in standardized sample preparation that allows the same parameter combination to be used between samples or experimenters.

To understand how experimenters assign foci, we chose Zip3-GFP and Msh4-GFP, two DNA interacting proteins that have been studied extensively in a range of organisms, including budding yeast as used here [36], [37], [38], [39], [40], [41]. In budding yeast, the number of Msh4 or Zip3 foci per nucleus have been used to estimate how many crossover depend upon these highly conserved proteins. Manual assignment of the same 21 images by three different experimenters showed tremendous variation in the number of foci assigned. Two ‘experts’ showed nearly a two-fold difference in their quantification of foci (Figure 1C). These differences are important because such differences have previously been attributed to different yeast strains and timing differences [36], [37]. Our data show that experimenters’ highly varied quantification could equally account for such differences in the published literature.

We identified four sources of variation in focus quantification (Figure 3). Of relatively minor contribution was P2 erroneously selecting non-maxima (one occurrence in the images assessed; Figure 3B). The two major contributing factors to variation were the interpretation of diffraction-limited foci as ‘doublets’ (Figure 3C), which could also have contributed to by double clicking (Figure 3A), and how the background level was selected (Figure 3D). We found that although P1 and P2 had similar total counts of foci (Figure 1C), this was due to selecting different foci. Specifically, P2 had set a higher background threshold compared to P1, but this potential deficit was offset by double-clicking and selecting doublets at a higher rate than P1. P3 had used the highest stringency of background threshold, interpreting a high proportion of fainter staining foci as noise (*e.g.* Figure 3D). This was particularly the case for dataset 2 (images 15–21), which included Image 21 where the Jaccard score was very low between experimenters P1 and P3 (<0.3).

The potential biological implications of these differences are important. For example, dataset 2 was concerned with the quantification of Msh4 foci. Msh4 has previously been reported to form foci in numbers that are equivalent to Zip3 and that colocalized with Zip3 on yeast chromosomes [37]. However, if faint Msh4 foci were excluded due to stringent threshold settings, then Msh4 focus numbers would be underestimated. Moreover, if the fainter Msh4 foci are of a different classification than the more intense ones that colocalize with Zip3, then the overall colocalization estimates would be inflated as well. This potential source of variation is critical, because the focus numbers and colocalization of Msh4 with Zip3 in yeast has been used to infer that Msh4 marks only sites of a subset of recombination events, *i.e.* those giving rise to reciprocal exchanges termed crossovers [37]. In contrast, Msh4 marks most sites of ongoing recombination in mammals and has been suggested to be important for general recombination processes as well as crossover recombination [39], [40]. Our findings that fainter staining foci are preferentially excluded due to variation in determining background noise by different experimenters (Figure 4, 5, 6) raise the possibility that Msh4 may also mark the majority of recombination sites in budding yeast. In summary, the significant variation in human assignment of foci raises the distinct possibility that our interpretation of biological processes more generally may be strongly biased to the first observer's characterization of the protein's behaviour. Once published, differences in or even reversal of conclusions would be difficult to facilitate leading at best to data loss and, at worst, to incorrect biological inferences that would be perpetuated in the field.

### FindFoci: an automated training algorithm closely matches human assignments and negates the need for manual, non-intuitive parameter selection

We reasoned that automating focus detection could not only speed up the time needed for quantification analysis, but could also greatly assist in eliminating inconsistencies. In many programmes, selecting a range of parameter choices for a single image and then applying this combination to other images in a batch or pipeline analysis is the norm [7], [10], [11], [42]. This requires expert knowledge of the parameters as well as substantial time in testing different parameter combinations. The search for optimal parameters is most often non-exhaustive and this may influence results, since parameter choices can affect the output of a focus detection algorithm. We developed an adaptable focus identification algorithm applicable to all images with local maxima (FindFoci) that closely matches human assignment on a variety of single images (Table 2). When trained on a single image, the algorithm gave average F1-scores of 94–97% when used to detect foci on the same image. To our knowledge, this is the first open-source programme that allows training of the algorithm as opposed to manual parameter configuration.

The FindFoci Optimiser (Figure 9) quickly searches parameter space and reports optimal parameter settings that closely match human assignments from approximately 20,000 combinations in ∼ 50 seconds per image. This is orders of magnitude faster than manual selection of parameters in applications such as CellProfiler. The automated warning that an optimal parameter is at an edge of the search space allows further refinement of the parameters. FindFoci thus reduces the parameter search time making it time efficient to optimise parameters for a pipeline analysis or to simply just record parameters for future reference.

Although the F1-scores were very high (Table 2), the optimised parameters obtained from single images do not exactly match the results of the manual assignment. This may be due to human error in the manual assignment (*e.g.* Figure 3B) but can also be due to failings in the algorithm, for example in the case of secondary spots that are not true local maxima due to overlap with a primary spot (see example Figure 3C). Cases will arise when the knowledge and experience of the scientist cannot be encapsulated easily within an analysis algorithm. If the algorithm fails to identify only a low percentage of foci selected manually by the scientist, then a semi-automated analysis is valid. In this case, the algorithm can be applied to the image as a first pass and then the results verified manually and additional spots added if necessary. Alternatively, the scientist may wish to label all spots manually but have the points aligned to the correct local maxima if available. To aid in this workflow we have created a helper application that analyses an image for all candidate maxima (Figure 9, FindFoci Helper). Existing points marked on the image are aligned to their nearest maximum. Additional points marked will be aligned to a maximum within a configured search radius. Points that cannot be assigned will be unmoved.

### Training across single images can skew subsequent data analysis

From most pipelines, it is unclear how a specific parameter combination has been selected for subsequent batch analysis. Even if optimal parameters are selected from a single image, the parameter combination derived from a single image may not improve performance across datasets within or between experimenters (Figure 7). In some cases, using a parameter combination from a single image gives rise to very low concordance (*e.g.* Image 21 from Experimenter P3 in Figure 7). Thus, although single parameter settings may work well for batch analysis in some cases [7], [11], this has to be empirically tested. For example, using single image parameters to assess a range of other images would be appropriate where the experimental work-up is very consistent and/or foci very bright and homogenous. We conclude that the use of analysis pipelines clearly speeds up the workflow, but does not necessarily improve performance and in some cases may significantly skew data analysis.

### Training FindFoci on multiple images improves consistency of focus analysis

We overcame the problem of single images skewing batch analysis (Figure 7) by training the algorithm on multiple images (Figure 8). Training on multiple images improved consistency in focus detection within experimenters (top row, Figure 8) and also improved the similarity in focus detection between experimenters.

From a practical point of view, how would an experimenter determine how many images to train the algorithm on before applying the parameter combination to a larger dataset? For a small dataset, the experimenter may want to manually mark all images and then use the algorithm to make parameter usage transparent. For larger datasets, the number of images the experimenter chooses to train the algorithm on would depend upon how representative the images are. Ideally, one would determine when outliers are reduced or eliminated (as in Figure 8), however, this is time consuming and requires expert data analysis. Figure 8 reveals that even when only a few images are used for training (n  =  5), outliers are relatively infrequent (less than 10% of the 100 combinations tested were outliers). More extreme outliers, where F1-scores were heavily affected (<0.4, *e.g.* P3 versus P1 and P3 versus P2), were eliminated when five or more images were used for training. If one desires a higher concordance where no outliers are acceptable, then one would choose a larger number of images, *e.g.* 11 in our case. In summary, if the dataset contains an image where the scoring of foci is atypical, using multiple images reduces its effect.

It is also possible to train the algorithm on the same images marked by two or more different experimenters. This will be subject to the caveat that the experimenters consistently mark similar foci. Our results show that the most intense foci are consistently selected by different experimenters, whereas the less intense foci are the subject of disagreement (Figure 4 and 5). If there is discordance between two experimenters, the final algorithm parameters will be an equal weighting between the two experimenters' styles of focus selection.

### Wider usage of FindFoci: assignment of nuclei and other subcellular structures

FindFoci can also be applied to a range of other biological applications. For example, automated nuclei counting in *Drosophila* embryos, *C. elegans*, and other multicellular organisms currently relies on ImageJ plugins such as the automated nuclei counter, ITCN. This and many other plugins require that the experimenter first measure nuclei diameter, distance between nuclei, and a range of other parameters in order to manually characterise the parameter describing the size range of the nucleus. FindFoci overcomes these tedious steps by allowing experimenters to simply click on the foci and use the algorithm to derive optimal parameters.

Figure 9 shows the automated workflow provided by the FindFoci software. Basic manual focus detection where images are labelled (‘Manual’) and foci counted manually can be facilitated using the FindFoci Helper, which aligns a clicked point on the image to the nearest local maximum. Alternatively, interactive manual focus selection can be performed using software such as the FindFoci GUI to get a labelled image. In this scenario, parameters can be manually adjusted to obtain labelled images and quantitative outputs. The FindFoci GUI is fast enough to allow real-time updates of the results, unlike other ImageJ plugins and CellProfiler.

In contrast to manual selection, our findings show that automated training allows the FindFoci algorithm to match human assignments. Thus, a few manually-labelled images can be used to train the FindFoci algorithm, using the FindFoci Optimiser. The initial range for input parameters can be determined using the FindFoci GUI to explore the effect of parameter changes on the identified foci. During training, the FindFoci Optimiser automatically reports parameters at the edge of their range, which can be readjusted to increase the search parameter space to find more optimal sets of parameter combinations. Unfortunately, the ImageJ/ImageJ2 architecture does not currently support executing programmes against multiple images. Therefore, we have developed a plugin that allows the FindFoci Optimiser to be executed against a directory containing multiple images and the results combined to provide parameter combinations derived from multiple training images. These parameters can then be used to set up a ‘batch analysis' (or pipeline) on a large number of images using the FindFoci Batch plugin. This process of marking five images, running the FindFoci Optimiser, and predicting foci on the 63 images took less than an hour. The rate determining step is training the FindFoci Optimiser. Therefore we implemented multi-threaded code so that multiple images can be processed in parallel to increase speed. FindFoci generates labelled images as well as extensive statistics on the foci identified.

All software is written as plugins for ImageJ/ImageJ2 and is available from our website (see list of downloads in Materials and Methods) or as an automated ImageJ2/Fiji update using the GDSC update site (http://fiji.sc/List_of_update_sites). The plugins are fully supported within the ImageJ macro recorder and can be used within scripts. FindFoci contains a built-in batch plugin that allows processing of all images within a directory using parameters loaded from file. In summary, FindFoci is fast and intuitive, enabling experimenters to explore parameter space interactively. It is able to match the focus assignment methods of individual experimenters closely (Table 2), thereby making the detection of foci transparent and parameterised. Importantly, concordance between experimenters improved using FindFoci compared to manual assignments and also performed better than using manually-optimised parameter settings in CellProfiler and thus, presumably FociCounter [11]. Finally, although we used 2D images, the algorithm can also be used for 3D stacks.

## Materials and Methods

### Image preparation

Strains Y2064 and Y2715 were induced to undergo meiosis as described previously [43]. Msh4-GFP or Zip3-GFP foci were detected with primary antibodies against GFP (guinea pig anti-GFP), and secondary antibodies using mouse FITC-conjugated antibodies as described previously. DNA was stained with DAPI. All conditions for spreading, detection, and imaging have been described previously [43]. All images are available in Dataset S1.

### Focus assignment and comparison

Manual focus assignments were made using the multi-point ROI tool in ImageJ. The experimenter was allowed to view the DAPI channel containing the DNA spread and the channel containing the foci. The experimenter was free to adjust contrast and other viewing controls as required. Following assignment the DAPI channel was thresholded using the Otsu method [44] and only ROI points within the mask were analysed. The same mask was used to filter foci from the automated methods to ensure a fair comparison of the foci of interest. Focus assignments were compared by iteratively assigning the closest pairs up to a radius of 8 pixels as matches. The remaining unmatched assignments from each set were counted. It took each of the experimenters more than one hour to analyse the 21 images.

### The FindFoci algorithm

The FindFoci algorithm first identifies potential foci and then expands these into peaks. The peaks are then merged to remove insignificant peaks. All steps can be controlled using configurable parameters.

Candidate foci are identified by selecting single pixels with intensities above all of their neighbours. All pixels with intensities below a background level are ignored. An allowance is made for foci that cover multiple pixels with the same intensity (plateau maximum) by positioning the candidate focus at the centre of mass of the plateau. The background level is set using thresholding methods, which determine the foreground pixels using an analysis of the intensity values in the image, or using an absolute intensity threshold.

Local maxima are expanded into peak regions by assigning surrounding (non-maximum) pixels to the appropriate maximum by following the uphill gradient. The expansion of each peak can be restricted relative to the height of the peak, *e.g.* expand the peak to any pixel within 50% of the maximum intensity. Following identification of peak regions the boundaries between peaks are calculated and the highest boundary points between touching peaks are stored as saddles.

A peak merge algorithm is then used to join insignificant, smaller peaks into their most significant neighbouring peak. In the case when a small peak has many suitable neighbours, the significant neighbour is defined using the highest saddle point. Peaks are identified as insignificant using their height and area as criteria. Peak merging is done in three stages. In the first stage, peaks are ranked by their highest saddle point. Each peak is checked using a height threshold and is merged if it is not distinct from the neighbour. Secondly, peaks are ranked by their size and merging is performed using total peak size. In the final stage peaks are ranked and merged using size above the highest saddle point. After every merge the size and highest saddle point for the new peak are updated. The final merge stage is optional since computation of the size above the new saddle point is expensive and reduces the algorithm's speed. By processing peaks in sorted order each merge is performed using a single pass over the data allowing fast elimination of insignificant peaks.

FindFoci allows noisy data to be smoothed using a Gaussian blur prior to peak identification. However, reported peak statistics always use the intensity values from the original, unsmoothed image. The algorithm can be applied to 2D or 3D images and is available as a plugin for ImageJ/ImageJ2. The plugin allows setting parameters to control the background level, search method, merge criteria and the display of results. The plugin is scriptable via the ImageJ/ImageJ2 macro facility and provides a GUI that allows the parameters to be adjusted with real-time update of the results. An optimiser is provided to identify the best parameters by comparing results to a reference image marked with foci of interest. The optimiser can use multiple reference images and combine the results across the dataset to identify the optimum combination of parameters.

### Focus alignment

Foci labelled on an image are reassigned to their closest true local maximum. The image background level is set using the minimum pixel value of the selected points minus the standard deviation of the image. This allows all maxima relevant to the selected points to be detected. Potential maxima in the image are assigned using the FindFoci algorithm with a minimum size of 1 pixel. Peaks are expanded and a mask is created from each maximum to label that region as belonging to the maximum. Foci to be assigned to local maxima are processed in descending order of pixel intensity. Each focus is assigned to the maximum using the region in which it resides. If the maximum is already assigned then the distance to the unavailable maximum is used as a search radius to locate the nearest unassigned maximum. If no maximum is available, the point will remain unaligned otherwise the point is moved to the assigned maximum.

### Training on multiple images

The FindFoci Optimiser was run using the same range of parameters on multiple images and the results for each image saved to file. The performance scores (Jaccard/F1-score) for each combination of parameters is aggregated across all the images and the best parameters selected using the best aggregated score. Before aggregation the scores for each image were optionally converted to allow fair comparison across images. The scores were converted using different methods: no conversion (raw score); the relative score; the z-score; or the rank. The relative score was computed for each image by identifying the best possible score for that image (top score) and then expressing the individual scores relative to the top score as a fraction (therefore the bottom score was assumed to be zero). The z-score was computed by subtracting the average score for the image and dividing by the standard deviation of the scores for the image. The rank was assigned by ordering the scores and labelling incrementally from 1. In the event of a tie between scores then the rank was assigned as equal for the set of values with the same score. Training using the raw score, relative score or z-score returned the same optimal parameters in our dataset. Training using the rank returned optimal parameters with lower performance.

Training across multiple images was performed using 1 to 21 images. The number of possible combinations was calculated using the formula n!/(r!(n-r)!). When the number of combinations was low (*e.g.* r = 1, combinations = 21; r = 2, combinations = 210) all combinations of images were enumerated to eliminate random sub-sample bias and duplicate combinations. When the number of combinations was higher than 210 (*e.g.* r = 3, combinations = 1,330; r = 11, combinations = 352,716) then a subset of combinations was produced by sampling randomly from the possible images; 100 random combinations were produced. Training using combinations of 1 to 21 images took approximately 45 minutes per experimenter on a single 2.67GHz CPU.

### CellProfiler

Analysis was performed using CellProfiler version 2.1 [10]. A range of manually selected parameters were tested for identifying foci. Small spots were enhanced using the EnhanceOrSuppressFeatures module using the Enhance Speckles option with a maximum feature size of 15–25 pixels. Foci were identified using the IdentifyPrimaryObjects module with an expected feature size range of min 2–5 and max 10–15 pixels, objects outside this range were optionally excluded. Global thresholding was performed using the Otsu or MoG methods and the objects optionally smoothed using a radius of 0–5 pixels and local maxima suppressed using a radius of 0–5 pixels.

### Statistics and plots

R was used to generate plots (ggplot2) [45] and carry out statistical analyses (www.r-project.org). The lmPerm package was used to conduct non-parametric ANOVA analysis [46]. Additional packages used were plyr, lattice, lme4, grid and MASS.

### List of Downloads

The FindFoci web page including a user manual describing all the plugins: http://www.sussex.ac.uk/gdsc/intranet/microscopy/imagej/findfoci

The FindFoci plugins are packaged within the GDSC ImageJ plugins and distributed using an ImageJ2/Fiji update site allowing simple install into ImageJ (http://fiji.sc/List_of_update_sites). Further details can be found here:

http://www.sussex.ac.uk/gdsc/intranet/microscopy/imagej/gdsc_plugins

The source code for the plugins is available on GitHub:

https://github.com/aherbert/GDSC

The GDSC ImageJ Batch Processing Guide contains help for creating batch analysis scripts in ImageJ:

http://www.sussex.ac.uk/gdsc/intranet/microscopy/imagej/batch

## Supporting Information

Dataset S1 **Dataset containing 21 images of spread, meiotic nuclei from budding yeast.** Foci were stained using fluorescently labelled antibodies against two DNA repair proteins: Zip3-GFP (images 1-14); and Msh4-GFP (images 15-21). The DNA was stained using DAPI.
(ZIP)
