# Supplementary figures and images for: FindFoci: A Focus Detection Algorithm with Automated Parameter Training That Closely Matches Human Assignments, Reduces Human Inconsistencies and Increases Speed of Analysis
Source: PLoS One. 2014 Dec 5;9(12):e114749. doi: 10.1371/journal.pone.0114749 (PMC4257716; doi:10.1371/journal.pone.0114749)

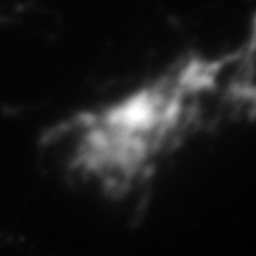

Supplement: Dataset S1 — Dataset containing 21 images of spread, meiotic nuclei from budding yeast. Foci were stained using fluorescently labelled antibodies against two DNA repair proteins: Zip3-GFP (images 1-14); and Msh4-GFP (images 15-21). The DNA was stained using DAPI. (ZIP) [file pone.0114749.s001.zip › ImageDataset/Image01_DNA.tif]

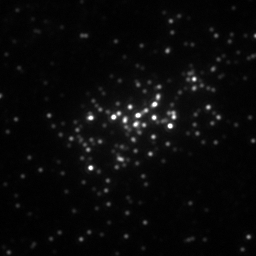

Supplement: Dataset S1 — Dataset containing 21 images of spread, meiotic nuclei from budding yeast. Foci were stained using fluorescently labelled antibodies against two DNA repair proteins: Zip3-GFP (images 1-14); and Msh4-GFP (images 15-21). The DNA was stained using DAPI. (ZIP) [file pone.0114749.s001.zip › ImageDataset/Image01_Foci.tif]

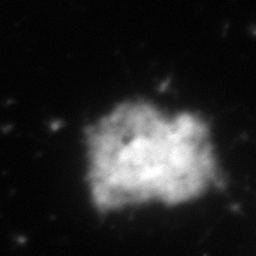

Supplement: Dataset S1 — Dataset containing 21 images of spread, meiotic nuclei from budding yeast. Foci were stained using fluorescently labelled antibodies against two DNA repair proteins: Zip3-GFP (images 1-14); and Msh4-GFP (images 15-21). The DNA was stained using DAPI. (ZIP) [file pone.0114749.s001.zip › ImageDataset/Image02_DNA.tif]

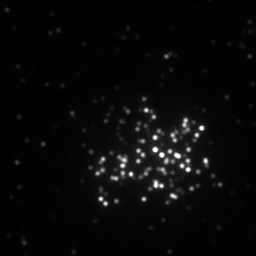

Supplement: Dataset S1 — Dataset containing 21 images of spread, meiotic nuclei from budding yeast. Foci were stained using fluorescently labelled antibodies against two DNA repair proteins: Zip3-GFP (images 1-14); and Msh4-GFP (images 15-21). The DNA was stained using DAPI. (ZIP) [file pone.0114749.s001.zip › ImageDataset/Image02_Foci.tif]

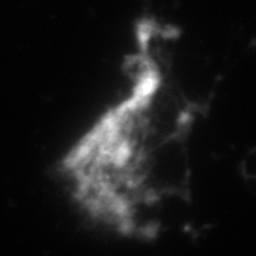

Supplement: Dataset S1 — Dataset containing 21 images of spread, meiotic nuclei from budding yeast. Foci were stained using fluorescently labelled antibodies against two DNA repair proteins: Zip3-GFP (images 1-14); and Msh4-GFP (images 15-21). The DNA was stained using DAPI. (ZIP) [file pone.0114749.s001.zip › ImageDataset/Image03_DNA.tif]

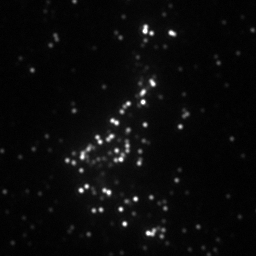

Supplement: Dataset S1 — Dataset containing 21 images of spread, meiotic nuclei from budding yeast. Foci were stained using fluorescently labelled antibodies against two DNA repair proteins: Zip3-GFP (images 1-14); and Msh4-GFP (images 15-21). The DNA was stained using DAPI. (ZIP) [file pone.0114749.s001.zip › ImageDataset/Image03_Foci.tif]

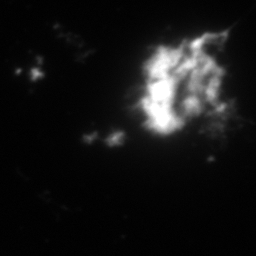

Supplement: Dataset S1 — Dataset containing 21 images of spread, meiotic nuclei from budding yeast. Foci were stained using fluorescently labelled antibodies against two DNA repair proteins: Zip3-GFP (images 1-14); and Msh4-GFP (images 15-21). The DNA was stained using DAPI. (ZIP) [file pone.0114749.s001.zip › ImageDataset/Image04_DNA.tif]

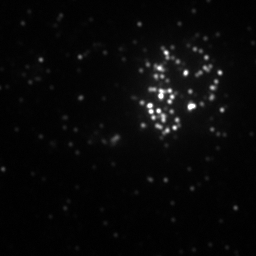

Supplement: Dataset S1 — Dataset containing 21 images of spread, meiotic nuclei from budding yeast. Foci were stained using fluorescently labelled antibodies against two DNA repair proteins: Zip3-GFP (images 1-14); and Msh4-GFP (images 15-21). The DNA was stained using DAPI. (ZIP) [file pone.0114749.s001.zip › ImageDataset/Image04_Foci.tif]

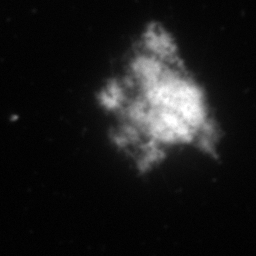

Supplement: Dataset S1 — Dataset containing 21 images of spread, meiotic nuclei from budding yeast. Foci were stained using fluorescently labelled antibodies against two DNA repair proteins: Zip3-GFP (images 1-14); and Msh4-GFP (images 15-21). The DNA was stained using DAPI. (ZIP) [file pone.0114749.s001.zip › ImageDataset/Image05_DNA.tif]

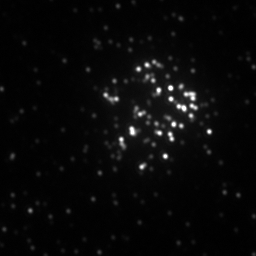

Supplement: Dataset S1 — Dataset containing 21 images of spread, meiotic nuclei from budding yeast. Foci were stained using fluorescently labelled antibodies against two DNA repair proteins: Zip3-GFP (images 1-14); and Msh4-GFP (images 15-21). The DNA was stained using DAPI. (ZIP) [file pone.0114749.s001.zip › ImageDataset/Image05_Foci.tif]

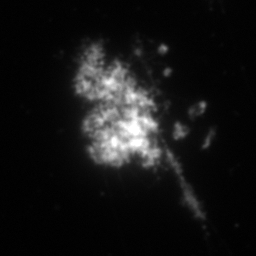

Supplement: Dataset S1 — Dataset containing 21 images of spread, meiotic nuclei from budding yeast. Foci were stained using fluorescently labelled antibodies against two DNA repair proteins: Zip3-GFP (images 1-14); and Msh4-GFP (images 15-21). The DNA was stained using DAPI. (ZIP) [file pone.0114749.s001.zip › ImageDataset/Image06_DNA.tif]

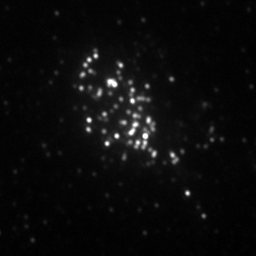

Supplement: Dataset S1 — Dataset containing 21 images of spread, meiotic nuclei from budding yeast. Foci were stained using fluorescently labelled antibodies against two DNA repair proteins: Zip3-GFP (images 1-14); and Msh4-GFP (images 15-21). The DNA was stained using DAPI. (ZIP) [file pone.0114749.s001.zip › ImageDataset/Image06_Foci.tif]

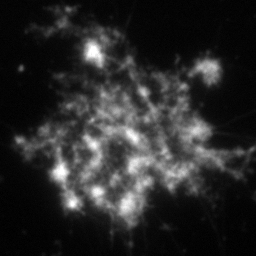

Supplement: Dataset S1 — Dataset containing 21 images of spread, meiotic nuclei from budding yeast. Foci were stained using fluorescently labelled antibodies against two DNA repair proteins: Zip3-GFP (images 1-14); and Msh4-GFP (images 15-21). The DNA was stained using DAPI. (ZIP) [file pone.0114749.s001.zip › ImageDataset/Image07_DNA.tif]

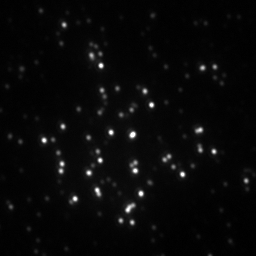

Supplement: Dataset S1 — Dataset containing 21 images of spread, meiotic nuclei from budding yeast. Foci were stained using fluorescently labelled antibodies against two DNA repair proteins: Zip3-GFP (images 1-14); and Msh4-GFP (images 15-21). The DNA was stained using DAPI. (ZIP) [file pone.0114749.s001.zip › ImageDataset/Image07_Foci.tif]

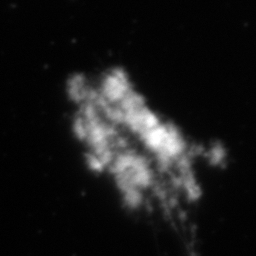

Supplement: Dataset S1 — Dataset containing 21 images of spread, meiotic nuclei from budding yeast. Foci were stained using fluorescently labelled antibodies against two DNA repair proteins: Zip3-GFP (images 1-14); and Msh4-GFP (images 15-21). The DNA was stained using DAPI. (ZIP) [file pone.0114749.s001.zip › ImageDataset/Image08_DNA.tif]

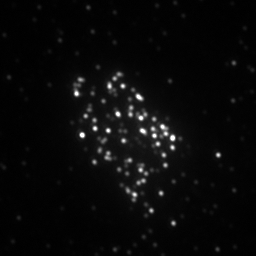

Supplement: Dataset S1 — Dataset containing 21 images of spread, meiotic nuclei from budding yeast. Foci were stained using fluorescently labelled antibodies against two DNA repair proteins: Zip3-GFP (images 1-14); and Msh4-GFP (images 15-21). The DNA was stained using DAPI. (ZIP) [file pone.0114749.s001.zip › ImageDataset/Image08_Foci.tif]

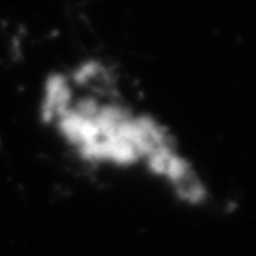

Supplement: Dataset S1 — Dataset containing 21 images of spread, meiotic nuclei from budding yeast. Foci were stained using fluorescently labelled antibodies against two DNA repair proteins: Zip3-GFP (images 1-14); and Msh4-GFP (images 15-21). The DNA was stained using DAPI. (ZIP) [file pone.0114749.s001.zip › ImageDataset/Image09_DNA.tif]

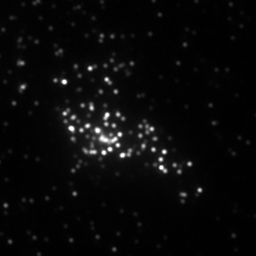

Supplement: Dataset S1 — Dataset containing 21 images of spread, meiotic nuclei from budding yeast. Foci were stained using fluorescently labelled antibodies against two DNA repair proteins: Zip3-GFP (images 1-14); and Msh4-GFP (images 15-21). The DNA was stained using DAPI. (ZIP) [file pone.0114749.s001.zip › ImageDataset/Image09_Foci.tif]

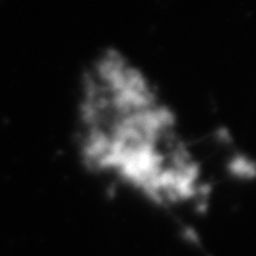

Supplement: Dataset S1 — Dataset containing 21 images of spread, meiotic nuclei from budding yeast. Foci were stained using fluorescently labelled antibodies against two DNA repair proteins: Zip3-GFP (images 1-14); and Msh4-GFP (images 15-21). The DNA was stained using DAPI. (ZIP) [file pone.0114749.s001.zip › ImageDataset/Image10_DNA.tif]

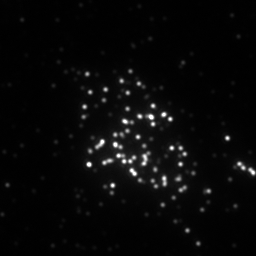

Supplement: Dataset S1 — Dataset containing 21 images of spread, meiotic nuclei from budding yeast. Foci were stained using fluorescently labelled antibodies against two DNA repair proteins: Zip3-GFP (images 1-14); and Msh4-GFP (images 15-21). The DNA was stained using DAPI. (ZIP) [file pone.0114749.s001.zip › ImageDataset/Image10_Foci.tif]

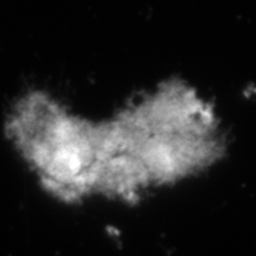

Supplement: Dataset S1 — Dataset containing 21 images of spread, meiotic nuclei from budding yeast. Foci were stained using fluorescently labelled antibodies against two DNA repair proteins: Zip3-GFP (images 1-14); and Msh4-GFP (images 15-21). The DNA was stained using DAPI. (ZIP) [file pone.0114749.s001.zip › ImageDataset/Image11_DNA.tif]

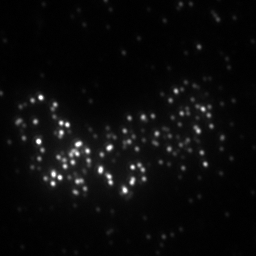

Supplement: Dataset S1 — Dataset containing 21 images of spread, meiotic nuclei from budding yeast. Foci were stained using fluorescently labelled antibodies against two DNA repair proteins: Zip3-GFP (images 1-14); and Msh4-GFP (images 15-21). The DNA was stained using DAPI. (ZIP) [file pone.0114749.s001.zip › ImageDataset/Image11_Foci.tif]

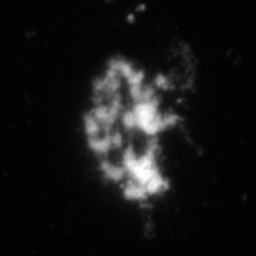

Supplement: Dataset S1 — Dataset containing 21 images of spread, meiotic nuclei from budding yeast. Foci were stained using fluorescently labelled antibodies against two DNA repair proteins: Zip3-GFP (images 1-14); and Msh4-GFP (images 15-21). The DNA was stained using DAPI. (ZIP) [file pone.0114749.s001.zip › ImageDataset/Image12_DNA.tif]

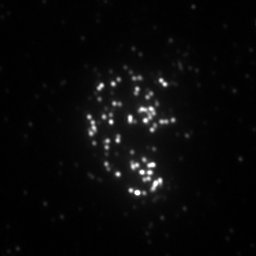

Supplement: Dataset S1 — Dataset containing 21 images of spread, meiotic nuclei from budding yeast. Foci were stained using fluorescently labelled antibodies against two DNA repair proteins: Zip3-GFP (images 1-14); and Msh4-GFP (images 15-21). The DNA was stained using DAPI. (ZIP) [file pone.0114749.s001.zip › ImageDataset/Image12_Foci.tif]

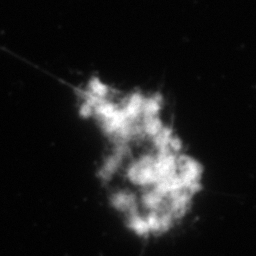

Supplement: Dataset S1 — Dataset containing 21 images of spread, meiotic nuclei from budding yeast. Foci were stained using fluorescently labelled antibodies against two DNA repair proteins: Zip3-GFP (images 1-14); and Msh4-GFP (images 15-21). The DNA was stained using DAPI. (ZIP) [file pone.0114749.s001.zip › ImageDataset/Image13_DNA.tif]

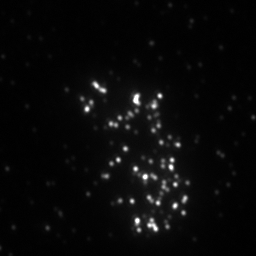

Supplement: Dataset S1 — Dataset containing 21 images of spread, meiotic nuclei from budding yeast. Foci were stained using fluorescently labelled antibodies against two DNA repair proteins: Zip3-GFP (images 1-14); and Msh4-GFP (images 15-21). The DNA was stained using DAPI. (ZIP) [file pone.0114749.s001.zip › ImageDataset/Image13_Foci.tif]

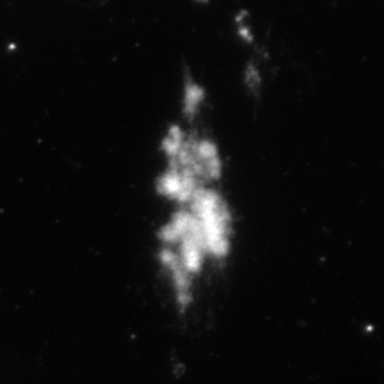

Supplement: Dataset S1 — Dataset containing 21 images of spread, meiotic nuclei from budding yeast. Foci were stained using fluorescently labelled antibodies against two DNA repair proteins: Zip3-GFP (images 1-14); and Msh4-GFP (images 15-21). The DNA was stained using DAPI. (ZIP) [file pone.0114749.s001.zip › ImageDataset/Image14_DNA.tif]

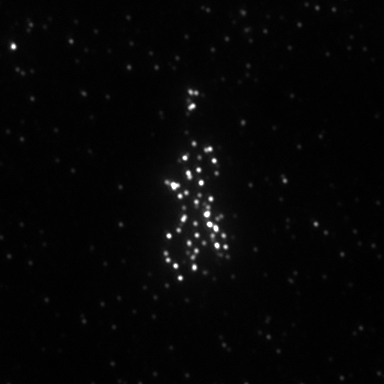

Supplement: Dataset S1 — Dataset containing 21 images of spread, meiotic nuclei from budding yeast. Foci were stained using fluorescently labelled antibodies against two DNA repair proteins: Zip3-GFP (images 1-14); and Msh4-GFP (images 15-21). The DNA was stained using DAPI. (ZIP) [file pone.0114749.s001.zip › ImageDataset/Image14_Foci.tif]

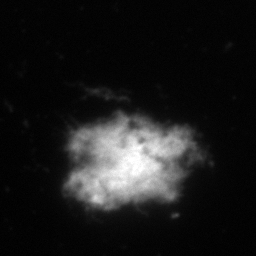

Supplement: Dataset S1 — Dataset containing 21 images of spread, meiotic nuclei from budding yeast. Foci were stained using fluorescently labelled antibodies against two DNA repair proteins: Zip3-GFP (images 1-14); and Msh4-GFP (images 15-21). The DNA was stained using DAPI. (ZIP) [file pone.0114749.s001.zip › ImageDataset/Image15_DNA.tif]

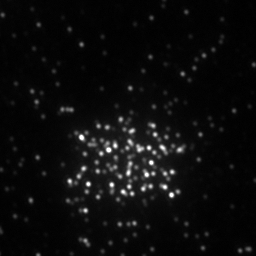

Supplement: Dataset S1 — Dataset containing 21 images of spread, meiotic nuclei from budding yeast. Foci were stained using fluorescently labelled antibodies against two DNA repair proteins: Zip3-GFP (images 1-14); and Msh4-GFP (images 15-21). The DNA was stained using DAPI. (ZIP) [file pone.0114749.s001.zip › ImageDataset/Image15_Foci.tif]

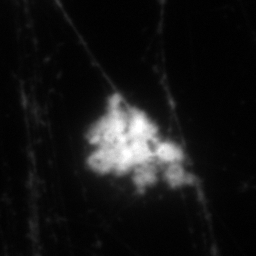

Supplement: Dataset S1 — Dataset containing 21 images of spread, meiotic nuclei from budding yeast. Foci were stained using fluorescently labelled antibodies against two DNA repair proteins: Zip3-GFP (images 1-14); and Msh4-GFP (images 15-21). The DNA was stained using DAPI. (ZIP) [file pone.0114749.s001.zip › ImageDataset/Image16_DNA.tif]

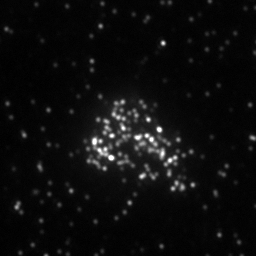

Supplement: Dataset S1 — Dataset containing 21 images of spread, meiotic nuclei from budding yeast. Foci were stained using fluorescently labelled antibodies against two DNA repair proteins: Zip3-GFP (images 1-14); and Msh4-GFP (images 15-21). The DNA was stained using DAPI. (ZIP) [file pone.0114749.s001.zip › ImageDataset/Image16_Foci.tif]

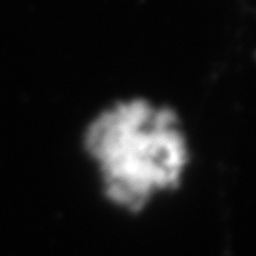

Supplement: Dataset S1 — Dataset containing 21 images of spread, meiotic nuclei from budding yeast. Foci were stained using fluorescently labelled antibodies against two DNA repair proteins: Zip3-GFP (images 1-14); and Msh4-GFP (images 15-21). The DNA was stained using DAPI. (ZIP) [file pone.0114749.s001.zip › ImageDataset/Image17_DNA.tif]

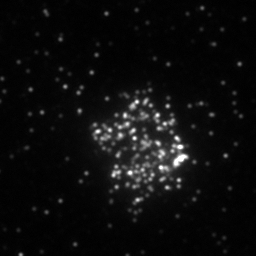

Supplement: Dataset S1 — Dataset containing 21 images of spread, meiotic nuclei from budding yeast. Foci were stained using fluorescently labelled antibodies against two DNA repair proteins: Zip3-GFP (images 1-14); and Msh4-GFP (images 15-21). The DNA was stained using DAPI. (ZIP) [file pone.0114749.s001.zip › ImageDataset/Image17_Foci.tif]

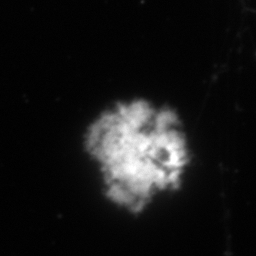

Supplement: Dataset S1 — Dataset containing 21 images of spread, meiotic nuclei from budding yeast. Foci were stained using fluorescently labelled antibodies against two DNA repair proteins: Zip3-GFP (images 1-14); and Msh4-GFP (images 15-21). The DNA was stained using DAPI. (ZIP) [file pone.0114749.s001.zip › ImageDataset/Image18_DNA.tif]

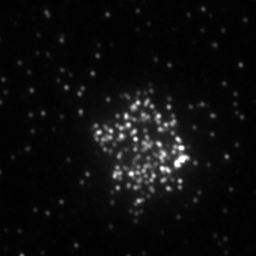

Supplement: Dataset S1 — Dataset containing 21 images of spread, meiotic nuclei from budding yeast. Foci were stained using fluorescently labelled antibodies against two DNA repair proteins: Zip3-GFP (images 1-14); and Msh4-GFP (images 15-21). The DNA was stained using DAPI. (ZIP) [file pone.0114749.s001.zip › ImageDataset/Image18_Foci.tif]

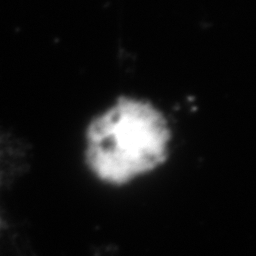

Supplement: Dataset S1 — Dataset containing 21 images of spread, meiotic nuclei from budding yeast. Foci were stained using fluorescently labelled antibodies against two DNA repair proteins: Zip3-GFP (images 1-14); and Msh4-GFP (images 15-21). The DNA was stained using DAPI. (ZIP) [file pone.0114749.s001.zip › ImageDataset/Image19_DNA.tif]

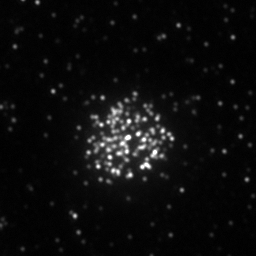

Supplement: Dataset S1 — Dataset containing 21 images of spread, meiotic nuclei from budding yeast. Foci were stained using fluorescently labelled antibodies against two DNA repair proteins: Zip3-GFP (images 1-14); and Msh4-GFP (images 15-21). The DNA was stained using DAPI. (ZIP) [file pone.0114749.s001.zip › ImageDataset/Image19_Foci.tif]

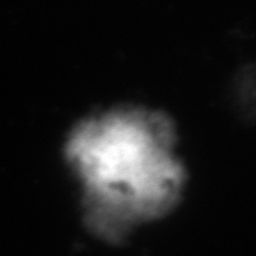

Supplement: Dataset S1 — Dataset containing 21 images of spread, meiotic nuclei from budding yeast. Foci were stained using fluorescently labelled antibodies against two DNA repair proteins: Zip3-GFP (images 1-14); and Msh4-GFP (images 15-21). The DNA was stained using DAPI. (ZIP) [file pone.0114749.s001.zip › ImageDataset/Image20_DNA.tif]

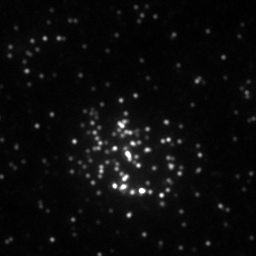

Supplement: Dataset S1 — Dataset containing 21 images of spread, meiotic nuclei from budding yeast. Foci were stained using fluorescently labelled antibodies against two DNA repair proteins: Zip3-GFP (images 1-14); and Msh4-GFP (images 15-21). The DNA was stained using DAPI. (ZIP) [file pone.0114749.s001.zip › ImageDataset/Image20_Foci.tif]

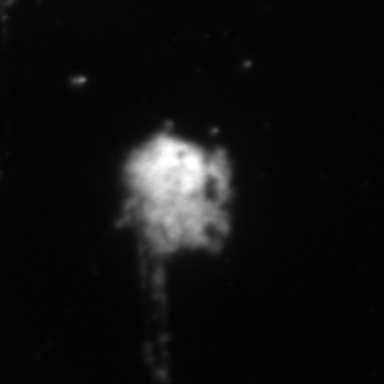

Supplement: Dataset S1 — Dataset containing 21 images of spread, meiotic nuclei from budding yeast. Foci were stained using fluorescently labelled antibodies against two DNA repair proteins: Zip3-GFP (images 1-14); and Msh4-GFP (images 15-21). The DNA was stained using DAPI. (ZIP) [file pone.0114749.s001.zip › ImageDataset/Image21_DNA.tif]

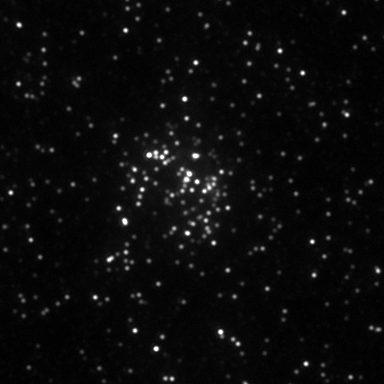

Supplement: Dataset S1 — Dataset containing 21 images of spread, meiotic nuclei from budding yeast. Foci were stained using fluorescently labelled antibodies against two DNA repair proteins: Zip3-GFP (images 1-14); and Msh4-GFP (images 15-21). The DNA was stained using DAPI. (ZIP) [file pone.0114749.s001.zip › ImageDataset/Image21_Foci.tif]
